# Home Enteral Nutrition in Patients with Cerebral Palsy in the Years 2012–2022: A Longitudinal Analysis of Data from the National Health Fund of Poland

**DOI:** 10.3390/nu16152394

**Published:** 2024-07-24

**Authors:** Maciej Zagierski, Angelika Górska, Agnieszka Zagierska, Joanna Augustyńska, Michał Seweryn, Agnieszka Szlagatys-Sidorkiewicz

**Affiliations:** 1 Department of Paediatrics, Gastroenterology, Allergology & Paediatric Nutrition, Medical University of Gdańsk, 80-210 Gdańsk, Poland; maciej.zagierski@gumed.edu.pl (M.Z.);; 2 Nutricia Polska Sp. z o.o., ul. Bobrowiecka 8, 00-728 Warsaw, Poland; 3 EconMed Europe, Młynska 9/4, 31-469 Krakow, Poland; j.augustynska@econmed.eu; 4 Faculty of Medicine and Health Sciences, Andrzej Frycz Modrzewski Krakow University, Gustawa Herlinga-Grudzinskiego 1, 30-705 Krakow, Poland

**Keywords:** cerebral palsy, enteral nutrition, home enteral nutrition, malnutrition, tube feeding

## Abstract

Background: Cerebral palsy (CP) often correlates with a higher risk of malnutrition, negatively affecting the quality of life of patients and their families. Enteral nutrition via a feeding tube should be considered to improve the nutritional status of CP patients. To date, there has been no nationwide registry of patients with CP in Poland. This study aimed to assess the prevalence of home enteral nutrition (HEN) provision in pediatric and adult patients with CP. Methods: We retrospectively analyzed data from the Polish National Health Fund (NFZ) on the provision of HEN in patients with CP in 2012–2022. A specially designed and validated questionnaire was sent to the 16 regional branches of NFZ. Results: Completed questionnaires were sent back from 12 NFZ branches. In 2022, CP cases increased by 7%, primarily among adults, while pediatric cases dropped by 21%. Despite a rising trend, the proportion of patients receiving HEN remained relatively low. Among children, it increased from 2.1% in 2012 to 3.3–3.5% in 2019–2021. For adults, it nearly doubled from 0.8% in 2012 to 1.7% in 2022. The prevalence of enteral feeding correlated with patient age, with a noticeable increase among older children and adolescents. Conclusions: National Health Fund data highlight the need for a nationwide registry of patients with CP. A relatively small proportion of pediatric and adult CP patients receive HEN. Increasing clinicians’ awareness of HEN availability is necessary to improve the quality of life for more CP patients.

## 1. Introduction

Cerebral palsy (CP) (or Little disease) is a group of lifelong conditions that affect a person’s ability to move and maintain posture. These motor and postural disorders are a consequence of irregular brain development during the fetal and neonatal period [1,2].

Cerebral palsy is the most common cause of disability among children worldwide. The diagnosis of CP is based on neurological examination, imaging studies, assessment of the child’s psychomotor development, and analysis of risk factors for CP. The diagnostic workup is usually difficult and time-consuming. Therefore, a correct diagnosis is often made at the age of 1–2 years or even later [3,4].

Cerebral palsy is the most common cause of motor disability among children [5]. Data on the prevalence and incidence of CP worldwide and in individual countries are limited and often come from studies conducted several years earlier. According to the Surveillance of Cerebral Palsy in Europe (SCPE), the mean incidence of CP over 10 years from 2002 to 2011 dropped from 1.9 to 1.6 per 1000 live births in both the subgroups of term and preterm infants [6]. Currently, it is estimated that there are 18,000,000 patients with CP in the world, of which 35% are adults (≥18 years of age) [6].

According to the most recent data, the number of CP patients in low-income and middle-income countries remains high. The birth prevalence of prenatal or perinatal CP is estimated at 3.4 per 1000 live births, whereas it is 1.5 per 1000 live births in high-income countries [7]. According to epidemiological data, the prevalence of CP in Poland is estimated at 2.0–2.5/1000 live births [3,8,9].

The most important and common symptom of CP is impaired postural and motor development. These may coexist with hearing and vision disorders, intellectual disability, seizure episodes (often epilepsy), pain, and difficulties in eating. The earlier the diagnosis is made, the sooner the patient will receive multispecialty care. Considering neuroplasticity, which is particularly strong during the developmental period, early initiation of therapy and rehabilitation can change the course of the disease. Fewer and/or milder complications can be expected, thus improving the quality and length of life of patients with CP [10].

Eating disorders were reported in more than half of the patients (53%) and, in some studies, even in up to 93% [11,12]. They are caused by multiple factors in patients with CP, including abnormalities in gross motor skills (inappropriate head position and difficulties in maintaining a stable head position) and fine motor skills (holding a glass or a spoon), as well as abnormal oral muscle strength, tension, and coordination. This not only affects the ability to eat on one’s own but also the possibility of being fed by a caregiver. As a result, patients with CP are at high risk of malnutrition [8,13,14]. Inadequate nutrient intake leads to chronic malnutrition, both qualitative and quantitative. According to various authors, malnutrition occurs in 40% to 94% of patients with CP and is related to both the type of the disease and the geographical as well as socio-behavioral factors [10,15,16]. Nutritional status, in turn, has important implications for the course of the disease and prognosis in terms of the quality and length of life. On the other hand, the severity of the disease and the degree of disability have an impact on the patient’s ability to consume food and, therefore, on nutrition [17]. If oral nutrition with appropriate meals (in terms of food consistency, etc.) does not ensure proper weight gain and/or weight maintenance, it is necessary to consider medical nutrition therapy. It may be either enteral nutrition or—in specific cases—parenteral nutrition.

Most patients with CP will require nutrition therapy at various stages of life. Depending on the timing of the nutritional intervention, it will aim at maintaining a normal nutritional status or at treating malnutrition. However, it should be underlined that maintaining the ability to take food orally is a priority. Moreover, oral nutrition is extremely important in terms of the psycho-emotional condition of the patient and their caregivers.

Enteral nutrition is applied by a feeding tube—either a nasogastric tube or a nasointestinal tube. In the long term, gastrostomy and jejunostomy should be considered as nonsurgical access for enteral nutrition. This method of nutrition is effective and safe, reducing the risk of choking [18,19].

Enteral feeding formulas remain the standard of care in patients with enteral access. Nutritionally complete, ready-to-use enteral tube feeds are suitable as a sole source of nutrition. The energy content, protein provision (short- or long-chain peptides), and specific lipid substrates (medium-chain triglycerides, omega-3 fatty acids) enable the dietary management of disease-related malnutrition in patients with malabsorption and/or maldigestion. This covers the patient’s requirement for nutrients both in terms of quality and quantity [20,21]. Enteral nutrition can be applied at home under medical guidance—either through a specialist clinic or a hospital unit. Home enteral nutrition (HEN) was reported to be significantly positively associated with improved quality of life of patients and their families [22,23,24].

One of the first studies on enteral nutrition in the Polish pediatric population showed an increase in the number of children on enteral nutrition by 21% during a 12-month follow-up (in 2010) [25]. In subsequent years, the number of children on enteral nutrition was consistently growing. More than 20% of these patients were children with neurological disorders, including 11.8% of patients with CP [26]. On the other hand, the number of adult patients on enteral nutrition increased by 42% in 2018. Patients with neurological disorders constituted more than half of this population, including 4.3% of patients with CP [27].

Importantly, in Poland, specific rules apply for the reimbursement of HEN. Only tube-fed patients with commercial enteral formulas are eligible for reimbursement, and there is no funding for oral nutritional support. In the pediatric population, commercial enteral formulas must constitute at least 51% of nutrition. However, while HEN has been available in Poland since 2007, there have been no registry studies of patients receiving this type of medical service. Moreover, there are no data on the number of patients with CP receiving enteral nutrition with an analysis according to age. Similarly, there is a lack of data regarding the direct indication for nutrition therapy [26,28]. Previous research only assessed the percentage of CP patients among patients who receive the guaranteed benefit of “home enteral nutrition” [25,26,27].

Considering the feeding disorders and a higher risk of malnutrition in patients with CP, this study aimed to assess the prevalence of HEN in pediatric and adult patients with CP in Poland.

## 2. Materials and Methods

This was a retrospective analysis of data from the Polish National Health Fund (Narodowy Fundusz Zdrowia [NFZ]) regarding the provision of HEN in the years 2012–2022. As the NFZ is the only payer of healthcare services in Poland, all medical procedures included in the analysis were covered by health insurance. The anonymized data were collected from the NFZ database; only epidemiologic statistics regarding the aggregated patient numbers were available. It was not possible to identify individual subjects.

To obtain data from the Polish public payer of healthcare services, a questionnaire was designed and validated, and in May 2023, it was sent to all 16 regional branches of the NFZ in Poland. Completed questionnaires were sent back by 12 branches from the following voivodeships: Lubelskie, Lubuskie, Łódzkie, Lesser Poland, Opolskie, Subcarpathian, Podlaskie, Pomeranian, Silesian, Świętokrzyskie, Greater Poland, and West Pomeranian.

The questionnaire requested data on the number of patients diagnosed with CP according to the following ICD-10 codes: G80 Cerebral palsy, G80.0 Spastic quadriplegic cerebral palsy, G80.8 Other cerebral palsy, and G80.9 Cerebral palsy, unspecified. The next part of the questionnaire concerned the number of CP patients divided into the healthcare services classified according to type: primary care, outpatient specialist care, hospital care, and rehabilitation care. The following age groups were also distinguished in the questionnaire: 0–3 years, 4–6 years, 7–12 years, 13–17 years, and 18 years or older.

In addition to general data on the number of patients with CP, the questionnaire aimed to collect data on the subpopulation of patients with CP who received HEN. HEN was defined in line with the nomenclature used by NFZ as follows: Home enteral nutrition (11.0000.048.02), Home enteral nutrition—services provided to persons aged 18 years or younger (services covered separately in PSZ [Hospital Security System for Healthcare Services]; 11.0000.602.02) or Home enteral nutrition (5.10.00.0000050) within the scope of Home parenteral and enteral nutrition (11.0000.042.02). For data on HEN, the same classification into subgroups was used as for the general data for CP.

Descriptive statistics for qualitative variables were presented as counts with percentages. The graphical representation of the descriptive data was also used. We built a trend line model to forecast the number of CP patients in the coming years. Different types of trend line models (linear, logistic, exponential) were tested, and the model that best fit the data according to the coefficient of determination (R^2^)—ranging from 0 (no fit) to 1 (perfect fit)—was selected.

Based on population data for the analyzed years, the prevalence ratio was calculated as the number of diagnosed and reported cases per 100,000 inhabitants. We considered the number of inhabitants in the 12 voivodeships and divided them into pediatric and adult patients.

Linear regression models were created to determine the possible future trends. For regression models, the analyzed data were considered a sample rather than a population. The results of the linear regression analysis were expressed as β coefficients with 95% confidence intervals (CIs). Two-sided tests were done with a *p*-value level of 0.05. The statistical analysis was conducted using R software (version 4.3.2).

## 3. Results

**Figure 1:**
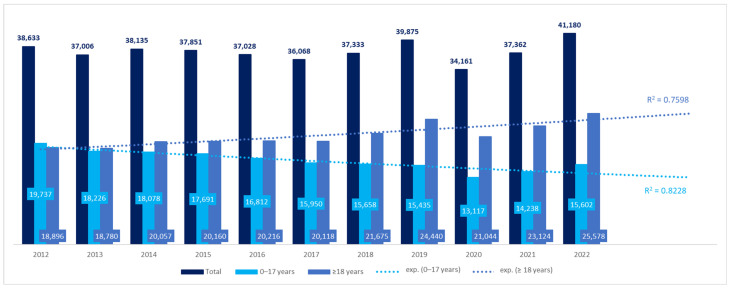
Number of patients with cerebral palsy in the years 2012–2022 (12 voivodeships) with trend lines.

We obtained data for 68.45% of the territory of Poland, inhabited by 68.8% of the Polish population in 2022. The number of patients with CP in the 12 voivodeships increased by 7%, from 38,633 in 2012 to 41,180 in 2022 (Figure 1). However, the difference was not significant (β coefficient: 59.91, 95%CI: −358.69–478.50, *p* > 0.05). The number of adult patients increased during the 11 years by 35%, while the number of pediatric patients with CP decreased by 21%. The differences in both groups were significant: β coefficient, 579.69, 95%CI: 327.83–831.55, *p* < 0.001 for adult patients, and β coefficient −519.78, 95%CI: −712.36 to −327.21, *p* < 0.001 for pediatric patients. Using a trend line model, the prognosis of the number of patients with CP in coming years was also estimated. Both in the pediatric and adult populations, the best fit was shown for the exponential trend line, according to the coefficient of determination (R^2^) (the equation for the pediatric population was y = 18,037*e^0.0268*x^ with R^2^ = 0.7598 and for the adult population: y = 19,708*e^−0.032*x^ and R^2^ = 0.8228). The number of adult patients with CP was estimated to reach 25,555 by 2025, while the number of children with CP—13,001 (Figure 1).

**Figure 2:**
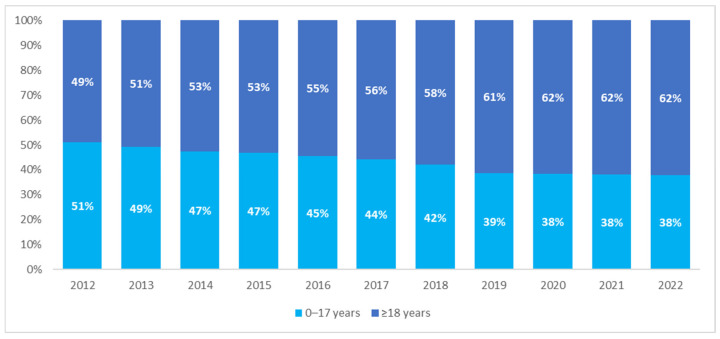
Percentage of pediatric and adult patients with cerebral palsy in the years 2012–2022.

The analysis of the age of patients with CP showed a comparable size of the pediatric and adult populations in 2012—children and adolescents aged 17 years or younger constituted 51% of the CP population. However, the structure of the population changed over the years, with the percentage of adults showing an increasing trend, from 49% in 2012 to 62% in 2022 (Figure 2).

**Figure 3:**
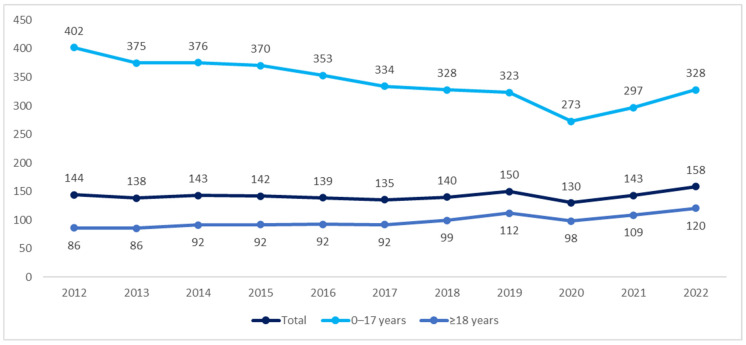
Incidence of cerebral palsy per 100,000 people in the years 2012–2022.

The incidence of CP in adult patients increased dynamically by 40%—from 86 per 100,000 people in 2012 to 120 per 100,000 people in 2022. In the pediatric population, the incidence decreased from 402 to 328 per 100,000 people in the years 2012–2022 (Figure 3). However, due to the coronavirus disease 2019 (COVID-19) pandemic in the years 2020–2021, the data for this period may be incomplete.

**Figure 4:**
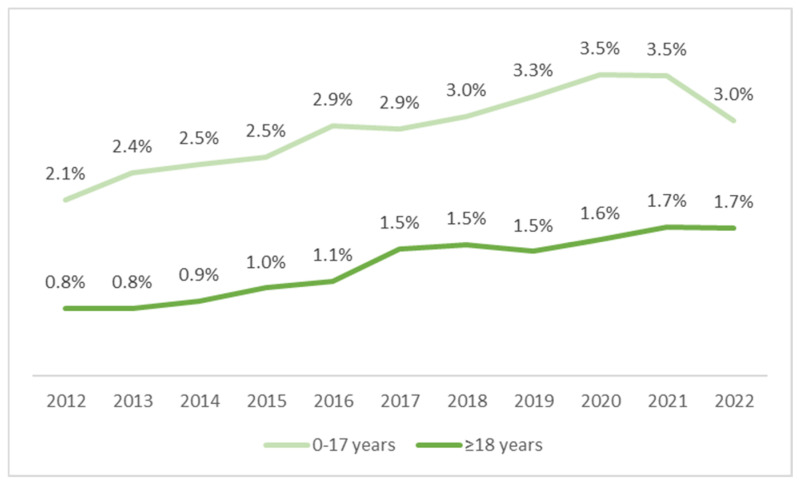
Percentage of pediatric and adult patients on enteral nutrition among all patients with cerebral palsy in the years 2012–2022.

The percentage of CP patients on enteral nutrition was low, although an increasing trend was observed, both in the pediatric and adult populations. In children, there was an increase from 2.1% (405/19,737) in 2012 to about 3.3–3.5% in the years 2019–2021, with a slight decrease to 3% in 2022 (465/15,602). However, this means an increase of 45% in 2022 as compared with 2012. In adult patients, an almost 2-fold increase was noted in the years 2012–2017 (from 0.8% to 1.5%). However, in subsequent years, the rate of growth slowed down, with an increase of 1.7% recorded in 2022. There was a systematic but moderate upward trend in the use of enteral nutrition in patients with CP (Figure 4).

**Figure 5:**
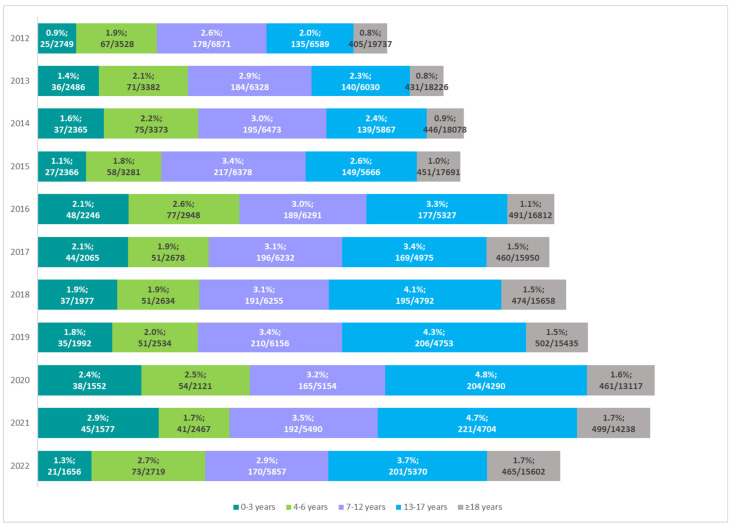
Number and percentage of patients on enteral nutrition among all patients with cerebral palsy in the years 2012–2022 by age group.

According to NFZ data, the frequency of enteral nutrition was associated with patient age. In recent years, there has been an increasing trend, especially in the group of older children (7–12 years) and adolescents (13–17 years). Until 2015, the highest percentage of CP patients on enteral nutrition was noted in the age group of 7–12 years. Among adolescents, the percentage of patients receiving enteral nutrition has increased from 2.6% in 2015 to 4.8% in 2020 (Figure 5).

**Figure 6:**
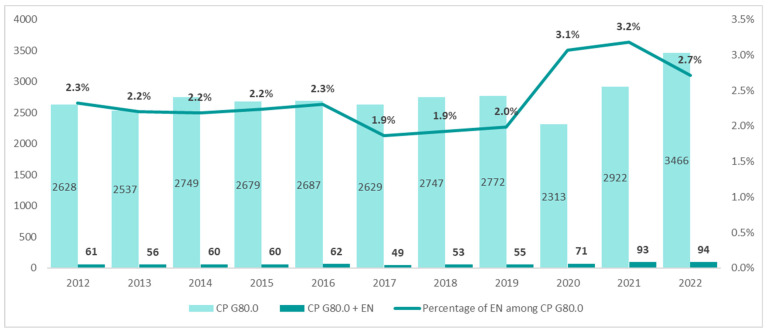
Number and percentage of patients on enteral nutrition (EN) among patients with spastic quadriplegic cerebral palsy (CP) (ICD-10 code G80.0) in the years 2012–2022.

The population of patients with the diagnosis of spastic quadriplegic CP (ICD-10 G80.0) in the years 2012–2021 was small and remained at a stable level of about 2628 patients. However, a significant increase to 3466 patients was noted in 2022. Until 2019, the annual number of patients receiving enteral nutrition was 61, but it increased by more than 50% in 2022 (94 patients) (Figure 6).

**Figure 7:**
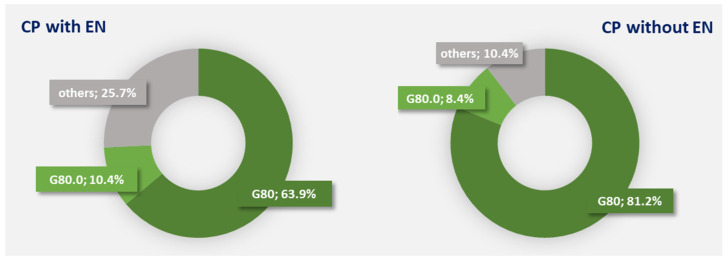
Percentage of patients with cerebral palsy (CP) according to ICD-10 diagnosis codes who received (**left-hand** panel) and did not receive (**right-hand** panel) enteral nutrition (EN) in 2022. G80—cerebral palsy, G80.0—spastic quadriplegic cerebral palsy, G80.8—other cerebral palsy, and G80.9—cerebral palsy, unspecified.

We also assessed the percentage of patients with spastic quadriplegic CP (ICD-10 code G80.0) in the group of patients receiving enteral nutrition and those not receiving enteral nutrition. In 2022, patients diagnosed with spastic quadriplegic CP constituted 10.4% of patients with CP on enteral nutrition vs. 8.4% of those not receiving enteral nutrition (Figure 7).

**Figure 8:**
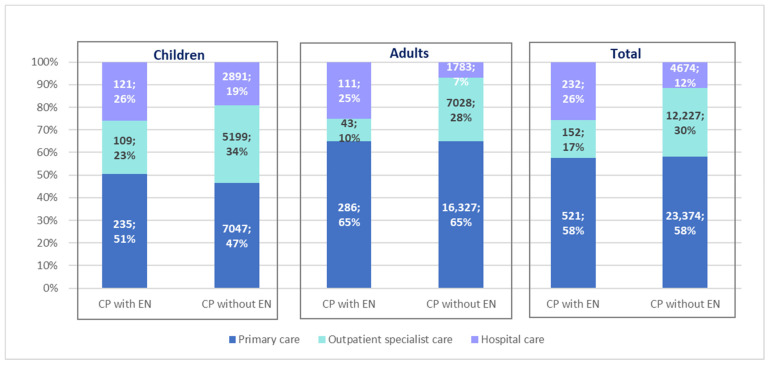
Number and percentage of patients with cerebral palsy (CP) receiving treatment as part of primary care, outpatient specialist care, and hospital care depending on the use of enteral nutrition (EN) in 2022.

The largest group of patients received treatment as part of primary care—every second patient used primary care services during the year, including both patients receiving and those not receiving enteral nutrition (Figure 8). Moreover, we observed that among patients not receiving enteral nutrition, 25% of patients using primary care services had the diagnosis of spastic quadriplegic CP (ICD-10 code G80.0).

**Figure 9:**
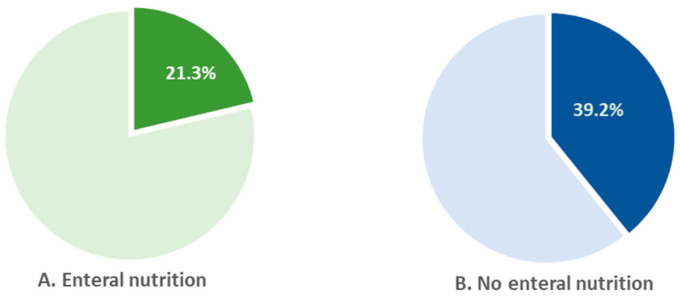
Percentage of children receiving rehabilitation care among children who received enteral nutrition (**A**) vs. those who did not receive enteral nutrition (**B**) in 2022.

In 2022, 39% of children who were not on enteral nutrition received rehabilitation care. In children on enteral nutrition, the percentage was almost 2-fold lower (21.3%) (Figure 9).

## 4. Discussion

In Poland, according to NFZ data, the number of people diagnosed with CP has increased during the years 2002–2012. Furthermore, the structure of this population has changed. In 2012, the number of children and adults with CP was similar, while 10 years later, more than 60% of the CP population were adult patients aged 18 years or older. In addition to the increase in this population, the number of patients with CP receiving HEN has grown.

The decreasing number of children with CP reflects the trend reported for developed countries [7]. Certainly, this is largely due to the rapid development of medicine and constant advances in perinatal care. According to Statistics Poland (Główny Urząd Statystyczny), life expectancy is increasing, and death rates are decreasing [9]. Thus, the life expectancy of patients with CP is also potentially increasing. Some studies indicated that around 22% of people with mild disability have a life expectancy similar to that of the general population. According to data from West Australia, the mortality of patients with severe CP has tended to shift from childhood to early adulthood [29]. Thus, with a stable incidence of CP and a higher survival rate of patients, the size of the adult population will increase markedly, and the number of pediatric patients will be similar or lower in subsequent years. The results of our study are in line with such a trend. The observed increase in the percentage of adults with CP receiving nutrition therapy is most likely the result of both pediatric to adult transition and adult patients being included in nutrition therapy ‘de novo’—the risk of complications and greater disability, including feeding difficulties, increases with the duration of the disease, so nutritional intervention becomes necessary. This hypothesis is consistent with previous research [27,28].

Of note, there was a comparable number of children with CP fed with enteral nutrition in the age groups of 0–3 years and 4–7 years, which only seemingly contradicts the above hypothesis.

We may suppose that some infants who received nutrition therapy as a service related to the primary diagnosis coded G80 in the ICD10 were diagnosed with a disease other than CP at the age of 4–7 years. Those children will not transit to the age group of 4–7 years, and the latter should be smaller in comparison to the younger group. However, the group of patients aged 4–7 years will be increased by patients with CP who require enteral nutrition only at this age.

In 2020, the number of patients with CP (in each age group) covered by the service of HEN was clearly lower than would be expected. This was the year of the pandemic caused by SARS-CoV-2, and there were 67,000 more deaths than in the previous year (2019). It was reported that SARS-CoV-2 was the cause of death in 43% of cases. Certainly, there were patients with CP in this group, although no data are available. During the pandemic, most of the healthcare services were provided remotely. As a result, the reporting of the services investigated in this study was not precise.

There were significant differences in the proportion of patients receiving enteral nutrition, depending on the type of the disease. Patients with spastic quadriplegic CP (ICD-10 G80.0) have the greatest need for early nutrition therapy, yet it is often delayed. In our study, the proportion of patients who received HEN among patients with spastic quadriplegic CP was slightly higher than among patients who did not receive it. However, this was not the most sizable group of patients on enteral nutrition, contrary to previous studies where the largest group of CP patients receiving nutrition therapy was those with the most severe disease [24,30]. Here, it is important to consider inaccuracies in ICD-10 coding, i.e., not always accurate coding and also different coding for a particular patient (own unpublished data). This error of precision is somewhat derived from the lack of a patient registry that would oblige healthcare professionals to use the ICD-10 codification carefully.

Each patient with CP undergoing physical therapy has an increased nutrient requirement and higher energy expenditure. Physical therapy is the most important form of therapy for the patient with CP, but many clinicians and caregivers believe that nutrition therapy via the nasogastric tube or gastrostomy makes motor rehabilitation impossible [31]. This misconception is often reinforced by the lack of widely available and accessible information on this type of treatment. It is important to address the insufficient knowledge and concerns of the patient and their caregivers by promoting enteral feeding not only as a form of treatment for severe malnutrition but, above all, as an effective means of preventing cachexia. Improved nutritional status, in turn, increases the effectiveness of rehabilitation.

The data used in this study were obtained from 12 voivodeships (75%), so they are incomplete and do not include information on the largest voivodeship with the largest population (i.e., Masovian voivodeship) or the most urbanized voivodeship (i.e., Masovian and Lower Silesian voivodeship).

Our results clearly show the uneven distribution of patients with CP across Poland and the uneven number of reported healthcare services of HEN for patients with CP. This is in line with previous research [25,26]. It does not seem possible that in some areas of Poland, there are no patients with CP or none of the patients receive nutrition therapy. It is likely that patients living in these “blank spots” on the map of Poland are treated in another voivodeship, and the services provided are reported according to the location of the center [25,26].

Despite the increasing number of patients with CP, including those receiving nutrition therapy, the percentage of patients receiving HEN as part of guaranteed health benefits is still low compared with other countries [27,28,32]. This may result from insufficient knowledge among healthcare professionals about the availability of this form of therapy. Moreover, there is a common belief that malnutrition in patients with chronic disease, especially those with motor impairment, is an inherent feature of the clinical picture of the underlying disease. It is the primary care personnel that are most responsible for preventing malnutrition, identifying feeding difficulties, and presenting available treatment options to the patient and their carers. Every effort should be made to increase knowledge about nutrition therapy by confronting beliefs with facts. Patient referral and initiation of nutrition therapy remain the responsibility of the nutrition clinic or hospital ward.

In this study, we additionally analyzed the forms of medical care that are used by patients with CP, both those receiving and not receiving HEN. It turns out that children are hospitalized more often than adults. It can be expected that adult patients, due to their biological age and disease duration, may have more complications and comorbidities (that require hospital treatment). Although most children’s diseases can usually be treated on an outpatient basis, we do not have information on the cause of the hospitalization. These can be hospital stays related to ongoing diagnostic workups but also to the insertion of a feeding tube and the start of nutrition therapy. Some procedures in children require anesthesia, which is not applicable in adults.

To our knowledge, this is the first such study in Poland. However, while it provides valuable data on HEN in Polish patients with CP, it is not without limitations.

First, the results of the study represent not more than 70% of the territory of Poland.

Secondly, data were obtained from the NFZ database, which includes services financed as part of primary care, outpatient specialist care, hospital care, and rehabilitation care provided to patients under public healthcare. The main source of funding within this system is the general compulsory health insurance in the NFZ. This means that we did not include services provided to patients as part of private healthcare.

Another weakness of this study is that the NFZ system, which was the source of the data used in this study, is a billing system. The database of billing products (so-called benefits) contains information on the products reported by the provider to the NFZ after a given medical service has been provided and on the medical procedures performed by the provider on a given patient. For this reason, there are some limitations to the data used. First, some patients may have been reported to the NFZ with different indications according to the ICD-10 code (e.g., in primary care, a patient is reported with the general code G80, while in inpatient care, a patient is defined by a more precise code, e.g., G80.0). In such a situation, the patient may have been included in the NFZ system twice. Also, the total number of patients treated as part of primary care, outpatient specialist care, and hospital care may be overestimated because, during the year, the services provided to a particular patient may be reported by different facilities. Similarly, the reporting of benefits by patient age may be overstated, as individual patients may change their age group during the year.

It should also be noted that our analysis did not include patients with CP who are under inpatient hospice care.

However, despite all these limitations, the NFZ database is the most reliable source of information from clinical practice on the use of medical services by insured patients in Poland.

Although there are many patients with CP in Poland, and there are numerous foundations and educational programs, no CP registries have been carried out, either for individual voivodeships or the whole country. Such registries make it possible not only to assess the actual incidence of the disease but also to monitor the prevalence and its trends.

It will help to create a multispecialized healthcare system for individual patients with CP. After the diagnosis is made, a designated coordinator will refer for all necessary consultations, procedures, and tests. All these services will be obligatory for all patients with CP and financed by the National Health Fund. This will prevent omission but also unnecessary, repeated procedures.

Taking into consideration the increasing number of CP patients, mostly adults, creating such a health program seems inevitable. The medical registry of CP patients is the cornerstone.

It inspired us to investigate how many national registries are conducted around the world. The results of the research were rather disappointing.

The first population-based registry of CP was introduced in 1950 in Denmark. Since then, numerous registries have been set up around the world to collect data from selected areas. The difficulties in maintaining nationwide registries are caused by several factors. In addition to official paperwork, it seems that ethnic differences can also play a significant role. This is reflected in the situation in the United States, where many registers are being kept but only within the individual states [33,34].

In response to these barriers, the SCPE was established in 1998 with the goal of creating a uniform database. Currently, the SCPE has 25 active centers in 20 European countries. However, these countries do not include Poland. As of 2023, the SCPE Common Database contains anonymized population data on more than 26,000 children with CP [35,36,37]. A comparative study of two databases from two continents, SCPE and the Australian Cerebral Palsy Register, seems to be promising in terms of global data on CP. The obtained results suggest that their major characteristics are similar [38]. This is perhaps another step towards the global unification of registries. On the other hand, the online survey included 45 CP registers (79% response rate) and spanned six continents: Africa, Asia, Australasia, Europe, North America, and South America. Twelve registries (27%) represented upper- or lower-middle-income countries, and there were no registries from low-income countries. It proved that 25% of identified items are collected by all registry networks [39].

Clearly, every effort must be made to ensure that the emerging registries meet specific and unified criteria so that the data collected can be compared and combined.

## 5. Conclusions

In conclusion, the currently available data on HEN for patients with CP in Poland are derived from the database of the public payer and are incomplete. With a stable incidence of CP and improved patient survival, the population of adult patients will continue to grow. To assess the full scale of the problem, it is mandatory to create a nationwide registry of patients with CP. Although an increasing number of people with CP receive enteral nutrition, there is still an insufficient number of CP patients covered by the guaranteed service of HEN. To improve the quality of life of more patients with CP, it is necessary to build awareness among clinicians about nutrition therapy and the guaranteed service of HEN. Propagating knowledge can take various forms (professional conferences, online courses, etc.). They should be addressed to healthcare providers of all specialties because CP patients are subject to multispecialist care. Various publications and handbooks dedicated to caregivers of patients will contribute to the increase in the number of patients on HEN. By these means, the quality of life of CP patients may improve.

## Data Availability

The original contributions presented in the study are included in the article, further inquiries can be directed to the corresponding author.
